# School pressure and psychosomatic complaints among Swedish adolescents: does physical activity play a buffering role?

**DOI:** 10.3389/fpubh.2024.1392999

**Published:** 2024-06-26

**Authors:** Alicia Birgersson, Jonas Landberg, Sara Brolin Låftman

**Affiliations:** Department of Public Health Sciences, Stockholm University, Stockholm, Sweden

**Keywords:** school pressure, psychosomatic complaints, physical activity, adolescents, Sweden

## Abstract

**Background:**

School pressure is a significant stressor in the lives of adolescents, recognised to be associated with psychosomatic complaints. Therefore, the exploration of potential buffering factors is a relevant task. This study aimed to examine the association between school pressure and psychosomatic complaints and the potentially moderating role of physical activity in a Swedish national sample of adolescents.

**Methods:**

Data were derived from the 2017/2018 Swedish Health Behaviour in School-aged Children (HBSC) survey, involving 3,745 participants aged 11–15 years. School pressure and physical activity were measured using single items. Psychosomatic complaints were assessed through an additive index based on the frequency of eight complaints. Covariates included gender, grade, and family affluence.

**Results:**

Linear regression analyses demonstrated a positive graded association between school pressure and psychosomatic complaints, while an inversely graded association was observed between physical activity and psychosomatic complaints. Physical activity did, however, not moderate the link between school pressure and psychosomatic complaints.

**Conclusion:**

Even though physical activity did not serve as a buffer, the direct effects of school pressure and physical activity on psychosomatic complaints suggest that supporting young people in managing school demands and promoting their engagement in physical activities could be effective measures in alleviating psychosomatic complaints.

## Introduction

Psychosomatic complaints can be defined as somatic and psychological health issues not attributed to any physical illness (1). Such complaints are reported by high shares of adolescents in many countries (2), including Sweden (3). Notably, in Sweden, the reporting among 13–15-year-olds has increased more than in any other Nordic country (4).

Research indicates a correlation between psychosomatic complaints and perceived stress, meaning that they can be interpreted and referred to as “stress-related” (5). One important stressor in the lives of adolescents is school pressure, defined as the imbalance between the demands imposed by the school and available resources to cope with them (6). Numerous studies have consistently shown a positive association between students’ experiences of school pressure and the occurrence of different mental health problems (7), including psychosomatic complaints (6, 8–12). Therefore, the exploration of factors that could act as buffers against this relationship is a relevant task (13).

One potential factor which may mitigate the association between school pressure and psychosomatic complaints is physical activity. Physical activity can be defined as anything ranging from light to vigorous exercise, and it is known to have beneficial effects on both physical (14–16) and mental health (17, 18). Physical activity is also related to adaptive coping strategies such as the positive reappraisal of stressors while persisting with a focus on the future (19). To date, however, only a limited number of studies have investigated the potentially buffering role of physical activity in the association between school pressure and psychosomatic complaints in adolescents, and findings are inconclusive. Haugland et al. (20) used data from the Norwegian Health Behaviour in School-aged Children (HBSC) study in 1997/1998 to assess the links between school pressure, physical activity, and psychosomatic complaints in 15-year-old students. They showed an association between school-related stress and psychosomatic complaints, and found that physical activity had a moderating role. More specifically, that the association between school-related stress and psychosomatic complaints was stronger among those with lower levels of physical activity compared to those with medium or higher levels of physical activity. A master thesis by Fugelsnes and Reiestad (21), based on data from the Norwegian Health HBSC study of 2017/2018, also reported that physical activity moderated the association between school stress and health complaints, indicating a buffering effect. However, the thesis did not find any moderating effect of physical activity on the association between school stress and life satisfaction (21). Another Norwegian study, conducted by Moksnes et al. (22), examined the links between stress and psychological functioning (anxiety, depression and self-esteem) in 13–18-year-olds but they did not find any moderating effect of leisure time on physical activity. Furthermore, in their study of 407 Swiss adolescents (mean age 14 years) from 2008, Gerber and Pühse (23) showed that school-based stress was associated with psychosomatic complaints, but physical activity did not serve as a moderator in this association.

In summary, the current body of research on school pressure, physical activity, and psychosomatic complaints is limited and offers inconclusive findings, underscoring the need for further studies. Additionally, considering that school-related factors have been recognised as one plausible explanation for the increase in psychosomatic complaints among adolescents in Sweden (4), investigating potential buffering factors within the Swedish context becomes highly relevant.

The aim of the present study was to examine the association between school pressure and psychosomatic complaints and the potentially moderating role of physical activity in a Swedish national sample of adolescents.

## Method

### Data and participants

Data were derived from the Swedish Health Behaviour in School aged Children (HBSC) survey of 2017/2018. The study is a cross-sectional survey conducted every fourth year among students in grade 5, 7 and 9, corresponding to ages 11, 13, and 15 years. It is carried out by the Public Health Agency of Sweden as part of an international WHO collaboration. For the 2017/2018 study, Statistics Sweden drew a nationally representative sample of schools across Sweden. First, schools were sampled, and thereafter, one class (in grade 5, 7, or 9) was randomly selected in each school. All students in the selected class were invited to participate. The survey was administered at school during school hours as a paper-and-pencil questionnaire. It was conducted anonymously, with students submitting their completed questionnaires in sealed envelopes to the teacher, who then sent them to Statistics Sweden. The response rate was 47% at the school level (comprising 213 schools) and 89% at the student level, and the total number of participants was 4,185 (24) (see Supplementary Table S1). For the current study, participants with missing information on any of the study variables were excluded, leading to a study sample of 3,745 participants (i.e., 89% of the total sample).

### Measures

Psychosomatic complaints were measured by the HBSC Symptom Checklist (20) including eight items: “How often have you in the past 6 months experienced the following: (a) headache, (b) stomachache, (c) backache, (d) felt low, (e) been irritated or in a bad mood, (f) felt nervous, (g) had difficulties falling asleep, (h) felt dizzy?” For each item, the response categories were (1) “About every day”, (2) “More than once a week”, (3) “About every week”, (4) “About every month”, and (5) “Rarely or never”. The eight items were reversely recoded and summed into a continuous scale between 8 and 40, with higher scores indicating greater psychosomatic complaints. The measure was constructed only for participants who had completed information about all eight symptoms. The internal consistency was high (Cronbach’s alpha = 0.83). The same measure has been used in previous studies to assess psychosomatic complaints in adolescents (6, 10, 20).

School pressure was measured by the question: “How pressured do you feel by the schoolwork you have to do?” The response categories were (1) “Not at all”, (2) “A little”, (3) “Some”, and (4) “A lot”. The same measure has been used in earlier studies to assess school-related stress in adolescents (8, 20, 25, 26).

Physical activity was measured by a question which read: “How often do you exercise on your leisure time outside of school so much that you are out of breath or sweating?” The response categories were (1) “Every day”, (2) “4–6 times a week”, (3) “2–3 times a week”, (4) “Once a week”, (5) “Once a month”, (6) “Less than once a month”, and (7) “Never”. Following the approach of Haugland et al. (20), three categories of physical activity were created; those who exercised less than once a month or less, 1–3 days a week, and 4–6 times a week or more often.

Sociodemographic factors such as gender, school grade, and family socioeconomic status have shown to be associated with both school pressure and psychosomatic complaints (9, 11, 20, 23, 27), rendering them important to consider. Hence, gender, grade and family affluence were used as control variables.

Gender included the categories “boy” and “girl”.

Grade included grades 5, 7, and 9, corresponding to ages 11, 13, and 15 years.

The Family Affluence Scale (FAS) was measured through a categorical index of six items pertaining to family socioeconomic status related to owning a car, having individual bedrooms, going on vacation, number of bathrooms in the home, having a dishwasher in the home, and having computers in the home (28, 29). In the current study, relative family affluence was used, distinguishing between students in the lowest 20% (low affluence), middle 60% (medium affluence) and top 20% (high affluence) in Sweden. A validation study has shown that the scale identifies low-and high-income households in Sweden (28).

### Statistical analysis

First, descriptive statistics were examined. Next, a series of linear regression models were conducted to analyse the associations between school pressure, physical activity, and psychosomatic complaints. Crude (unadjusted) models included one independent variable at a time to assess their bivariate associations with psychosomatic complaints. Model 1 included school pressure, gender, grade, and family affluence. Model 2 added physical activity. To determine the potentially moderating effect of physical activity in the association between school pressure and psychosomatic complaints, we included an interaction term using Stata’s factorial command (#). To assess if the interaction term was statistically significant, the model with the interaction term was compared to the model without the interaction term using a Wald test. Finally, to further explore the links between school pressure, physical activity and psychosomatic complaints, we performed regression analyses of school pressure and psychosomatic complaints stratified by the level of physical activity, containing students who reported to be physically active (a) once a month or less; (b) 1–3 days a week; or (c) 4 days a week or more. To account for the fact that students were clustered in classes, robust standard errors were estimated. The number of classes was 213. All statistical analyses were performed using Stata, version 17 (30).

### Ethics approval and consent to participate

Ethical review and approval were not required for the study on human participants in accordance with the local legislation and institutional requirements. See information provided by the Swedish Ethical Review Board: https://etikprovningsmyndigheten.se/en/what-the-act-says/ The Swedish Health Behaviour in School-aged Children (HBSC) dataset does not include any personal identification information. The questionnaire is filled out by students anonymously and voluntarily. Since the study does not include personal identification information involve the collection of sensitive data, it did not require formal approval from an ethical review board. Informed consent was obtained from the participating students. Schools informed parents/guardians about the upcoming study, and parents who preferred their children not to take part were requested to notify the school. All methods were performed in accordance with the relevant guidelines and regulations.

### Large language models

ChatGPT was used to check the grammar, proofread the text and provide clarifications.

## Results

Descriptive statistics are presented in Table 1. In the study sample, 13.8% reported feeling “not at all” pressured by school, while 44.9% reported feeling “a little” pressure, 25.5% reported feeling “some” pressure, and 15.8% reported feeling “a lot” of school pressure. Whereas 15.1% of the study sample exercised once a month or less, 48.7% exercised 1–3 days a week, and 36.2% exercised 4 days a week or more. The sample consisted of 48.8% boys and 51.2% girls. The distribution across grades was somewhat uneven with 26.7% in grade 5, 33.7% in grade 7, and 39.6% in grade 9. The categories of relative family affluence comprised 15.1% (lowest 20 pct), 67.8% (medium 60 pct), and 17.1% (highest 20 pct) of the study sample. The mean score for psychosomatic complaints was 18.69. The distributions of physical activity, gender, grade, family affluence and psychosomatic complaints by the main exposure, i.e., school pressure, are presented in the Supplementary Table S2.

**Table 1:** Descriptives of the study sample.

	*n*	%
School pressure		
Not at all	518	13.8
A little	1,683	44.9
Some	954	25.5
A lot	590	15.8
Physical activity		
Once a month or less	565	15.1
1–3 days a week	1,825	48.7
4 days a week or more	1,355	36.2
Gender		
Boys	1,827	48.8
Girls	1,918	51.2
Grade		
5	1,001	26.7
7	1,262	33.7
9	1,482	39.6
Relative family affluence		
Lowest 20pct	567	15.1
Medium 60pct	2,539	67.8
Highest 20pct	639	17.1

	Mean	S.D.
Psychosomatic complaints	18.69	6.71

*n* = 3,745.

Subsequently, a series of linear regression analyses were performed with psychosomatic complaints as the outcome (Table 2). The crude analyses showed that greater school pressure was associated with higher levels of psychosomatic complaints in a graded pattern. Girls reported higher levels of psychosomatic complaints than boys. Furthermore, psychosomatic complaints increased with grade. There were no statistically significant differences in psychosomatic complaints by relative family affluence. Physical activity was inversely associated with psychosomatic complaints. In Model 1, including school pressure, gender, grade, and family affluence, the estimates were attenuated but remained statistically significant, with the exception of grade that turned non-significant. Model 2 added physical activity, which was significantly associated with psychosomatic complaints also when adjusting for school pressure and the covariates. Finally, an interaction term between school pressure and physical activity was added, but this was not statistically significant (*p* = 0.073). We also tested interactions between gender and all other variables (not presented in Table). The interaction terms for gender and school pressure, as well as gender and grade, reached statistical significance (both at *p* < 0.001), while those for gender and family affluence, as well as gender and physical activity, did not (*p* = 0.562 and *p* = 0.273, respectively). Gender stratified analyses are presented in the Supplementary Tables S3, S4.

**Table 2:** Results from linear regression analyses of psychosomatic complaints regressed on school pressure, physical activity, and covariates.

	Crude^a^		Model 1^b^		Model 2^c^	
	b	95% CI	b	95% CI	b	95% CI
School pressure						
Not at all (ref.)	0.00	–	0.00	–	0.00	–
A little	2.74***	2.08, 3.39	2.43***	1.75, 3.10	2.43***	1.76, 3.10
Some	6.09***	5.41, 6.77	5.32***	4.57, 6.08	5.31***	4.56, 6.06
A lot	9.22***	8.42, 10.03	8.26***	7.38, 9.13	8.21***	7.34, 9.09
Gender						
Boys (ref.)	0.00	–	0.00	–	0.00	–
Girls	3.47***	3.01, 3.92	2.34***	1.91, 2.76	2.27***	1.85, 2.69
Grade						
5 (ref.)	0.00	–	0.00	–	0.00	–
7	1.12**	0.43, 1.81	−0.05	−0.64, 0.54	−0.06	−0.64, 0.53
9	2.86***	2.17, 3.55	0.31	−0.32, 0.95	0.30	−0.34, 0.94
Relative family affluence						
Lowest 20pct (ref.)	0.00	–	0.00	–	0.00	–
Medium 60pct	0.11	−0.55, 0.77	−0.01	−0.57, 0.54	0.11	−0.45, 0.67
Highest 20pct	−0.65	−1.47, 0.18	−0.47	−1.15, 0.22	−0.24	−0.93, 0.45
Physical activity						
Once a month or less (ref.)	0.00	–			0.00	–
1–3 days a week	−1.46***	−2.13, −0.80			−0.83**	−1.40, −0.25
4 days a week or more	−2.07***	−2.80, −1.34			−1.11**	−1.74, −0.47
School pressure # Physical activity^d^						*p* = 0.073

Regression coefficients (b) and 95% confidence intervals (95% CI) with robust standard errors. *n*=3,745. ***p < 0.001; **p < 0.01; *p < 0.05.

a Crude analyses include one independent variable at the time.

b Model 1 includes school pressure, gender, grade, and relative family affluence.

c Model 2 includes school pressure, gender, grade, relative family affluence, and physical activity.

d *p*-value from Wald test comparing the model fit between models without and with an interaction term between school pressure and physical activity.

**Figure 1:**
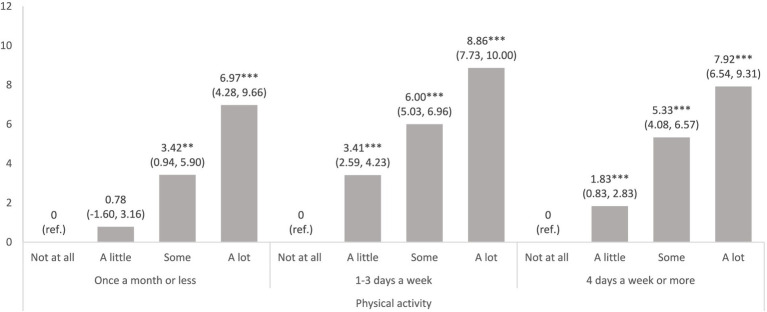
Results from three separate linear regression analyses of psychosomatic complaints regressed on school pressure, stratified by categories of physical activity. Models adjusted for gender, grade, and relative family affluence. Regression coefficients (b) and 95% confidence intervals (95% CI) with robust standard errors. ***p < 0.001; **p < 0.01; *p < 0.05.

To further scrutinise the non-significant interaction between school pressure and physical activity, we examined the association between school pressure and psychosomatic complaints stratified by the level of physical activity. The findings, depicted in Figure 1, illustrate a graded, positive association between school pressure and psychosomatic complaints, with a similar pattern observed irrespective of the level of physical activity.

## Discussion

The current study aimed to examine the association between school pressure and psychosomatic complaints as well as the potentially moderating role of physical activity. The results showed a clear positive graded association between school pressure and psychosomatic complaints, similar to what has been found in prior studies based on HBSC data from different countries and waves (6, 8, 10, 11, 20, 21) and on Swedish local datasets utilising different measures (9, 12), thus confirming the role of school pressure as a significant stressor. Physical activity did not, however, buffer against the association between school pressure and psychosomatic complaints. The interaction term examining the potential moderating effect of physical activity was not statistically significant. Additionally, analyses stratified by physical activity level revealed that the relationship between school pressure and psychosomatic complaints remained consistent in magnitude across different levels of physical activity.

The lack of a statistically significant buffering effect of physical activity aligns with the results of Moksnes et al.’s study among Norwegian adolescents (22) and Gerber and Pühse’s study among Swiss adolescents (23), but contradicts the findings of Haugland et al. (20) and those of Fugelsnes and Reiestad (21). This may appear somewhat unexpected given that our study utilised similar or identical measures as those in Haugland et al. (20) and Fugelsnes and Reiestad (21), and was also conducted within a Scandinavian context. One notable difference is, however, that Fugelsnes and Reiestad’s study utilised another measure of physical activity, which was based on the number of days of at least 60 min of physical activity during the past 7 days. Notwithstanding, it should be noted that schools in Sweden have undergone numerous reforms and significant changes over the past decades (31), coinciding with a reported increase in the prevalence of psychosomatic complaints among adolescents (3, 24). Indeed, the Public Health Agency of Sweden has argued that school factors are likely to be one explanation behind the increase in psychosomatic complaints (4). Comparing data from the Swedish and the Norwegian HBSC of 2017/2018 shows that fewer Swedish than Norwegian students report to be satisfied with school (26). Furthermore, the proportion of students who reported high school pressure but not high school satisfaction was larger in Sweden than in Norway (26). It is possible to speculate that the buffering effects of physical activity are less likely to occur when students experience school pressure and are simultaneously dissatisfied with school in general.

While our results align more closely with those of Moksnes et al. (22) and Gerber and Pühse (23), indicating no moderating effect of physical activity, it is important to note that there are differences between our study and these prior studies. For instance, Moksnes et al. (22) used a broader measurement of stress and also examined different outcomes, in terms of state depression, state anxiety and self-esteem. Their measure of physical activity referred to assessing how many days per week the participants were engaged in at least 20 min of physical activity (22). Likewise, in the study by Gerber and Pühse, physical activity was measured with a single item assessing how many days in the last week the participants were engaged in at least 20 min of physical activity (23).

Despite the absence of a statistically significant buffering effect of physical activity on the association between school pressure and psychosomatic complaints, the results of the present study indicate that higher levels of physical activity are associated with fewer psychosomatic complaints in general. This association holds true regardless of the level of school pressure. This finding reflects earlier research which has shown positive effects of physical activity on both physical (14–16) and mental health (17, 18). Additionally, physical activity has been shown to be related to certain adaptive coping strategies in adolescents, which may lead to less stress and psychosomatic complaints (19). Thus, it is important that parents and teachers are encouraged to help students engage in organised physical activity outside of school hours. Such interventions have shown to increase the likelihood of reaching the recommended amount of daily exercise (32). The clear associations between school pressure and psychosomatic complaints underscore the significance of providing support to adolescents in coping with the demands imposed on them in school (13). Finally, despite the absence of a statistically significant buffering effect in the current study, it remains plausible that physical activity could serve as a buffer against other stressors and/or mitigate other adverse health outcomes.

### Strengths and limitations

The main merit of the current study is the data material, which was based on a nationally representative sample of schools in Sweden, and with a high response rate at the student-level (89%). However, the lower participation rate at the school-level (47%) can be regarded as a constraint. The possible systematic bias in the non-response among students should also be acknowledged. For instance, it is plausible that students with high levels of psychosomatic complaints were more likely to be absent on the day that the survey was conducted. Nevertheless, such possible bias is more likely to have resulted in an underestimation of the associations rather than an overestimation. Additionally, there was internal non-response. However, when comparing the variable distributions in the study sample (Table 1) with those in the total sample (Supplementary Table S1), no substantial differences were observed.

One limitation pertains to the use of single-item measures for assessing school pressure and physical activity, which may increase the risk of misclassification and self-reporting bias (20). In contrast, psychosomatic complaints were assessed using an index based on eight items. While psychosomatic complaints are recognised indicators of stress (5), it is important to acknowledge that reporting may be influenced by various medical and non-medical factors. We cannot determine if certain health complaints reflect underlying medical conditions. However, the additive index considers both the number and frequency of symptoms, which mitigates the likelihood that high levels of psychosomatic complaints are solely driven by specific symptoms that may be expressions of medical conditions.

Finally, it is important to acknowledge that due to the cross-sectional nature of the data, the question of causality cannot be approached with support in the data. To study school pressure in relation to psychosomatic complaints and physical activity in a more causal capacity, it would be appropriate to apply a longitudinal design (20). Relatedly, the possibility of reverse causality cannot be ruled out (33). It could be the case that students with psychosomatic complaints may have experienced greater school pressure or avoided physical activity because they already suffered from health issues. The possibility of unmeasured confounders should also be acknowledged. While the analyses accounted for gender, grade, and family affluence, it is possible that other factors, e.g., psychosocial adversities in the family, may have influenced the associations (27).

## Conclusion

The findings of this study indicate that while physical activity alone did not act as a buffer, both school pressure and physical activity had direct effects on psychosomatic complaints. This suggests that assisting young individuals in coping with academic stressors and encouraging their participation in physical activities could be beneficial strategies for reducing psychosomatic complaints.

## Data availability statement

The datasets presented in this study can be found in online repositories. The names of the repository/repositories and accession number(s) can be found at the HBSC Data Management Center, University of Bergen. Please see: https://www.uib.no/en/hbscdata/113290/open-access.

## Ethics statement

Ethical approval was not required for the study involving humans in accordance with the local legislation and institutional requirements. The studies were conducted in accordance with the local legislation and institutional requirements. Written informed consent to participate in this study was not required from the participants in accordance with the national legislation and the institutional requirements.

## Author contributions

AB: Conceptualization, Formal analysis, Writing – original draft. JL: Supervision, Writing – review & editing. SL: Supervision, Writing – review & editing.
