# Supplementary material for: School pressure and psychosomatic complaints among Swedish adolescents: does physical activity play a buffering role?
Source: Front Public Health. 2024 Jun 26;12:1392999. doi: 10.3389/fpubh.2024.1392999 (PMC11233534; doi:10.3389/fpubh.2024.1392999)
Supplement: Supplementary file 1 [file Data_Sheet_1.docx]

**Supplementary Material**

**Table S1** Descriptive statistics of the full sample. n=4,185

|  | n | % |
| --- | --- | --- |
| School pressure |  |  |
| Not at all | 581 | 14.3 |
| A little | 1,839 | 45.2 |
| Some | 1,020 | 25.1 |
| A lot | 631 | 15.5 |
| *Missing* | *114* | *-* |
| Physical activity |  |  |
| Once a month or less | 614 | 15.2 |
| 1-3 days a week | 1,963 | 48.6 |
| 4 days a week or more | 1,460 | 36.2 |
| *Missing* | *148* | *-* |
| Gender |  |  |
| Male | 2,081 | 49.7 |
| Female | 2,104 | 50.3 |
| *Missing* | *0* | *-* |
| Grade |  |  |
| 5 | 1,174 | 28.1 |
| 7 | 1,423 | 34.0 |
| 9 | 1,588 | 37.9 |
| *Missing* | *0* | *-* |
| Relative family affluence |  |  |
| Lowest 20pct | 628 | 15.7 |
| Medium 60pct | 2,688 | 67.0 |
| Highest 20pct | 696 | 17.3 |
| *Missing* | *173* | *-* |
|  | Mean | s.d. |
| Psychosomatic complaints (n=4,009) | 18.63 | 6.68 |
| *Missing (n=176)* | - | - |

**Table S2** Distributions of physical activity, gender, grade, family affluence and psychosomatic complaints by school pressure, assessed with chi-square tests and ANOVAs. n=3,745

|  | School pressure | | | |  |
| --- | --- | --- | --- | --- | --- |
|  | Not at all | A little | Some | A lot | χ^2^ |
| Physical activity |  |  |  |  |  |
| Once a month or less | 12.4 | 38.1 | 28.1 | 21.4 |  |
| 1-3 days a week | 13.5 | 47.0 | 24.6 | 14.9 |  |
| 4 days a week or more | 14.9 | 45.0 | 25.5 | 14.5 | 25.59*** |
| Gender |  |  |  |  |  |
| Male | 18.7 | 50.4 | 20.5 | 10.5 |  |
| Female | 9.2 | 39.8 | 30.2 | 20.8 | 183.55*** |
| Grade |  |  |  |  |  |
| 5 | 27.4 | 54.4 | 13.9 | 4.4 |  |
| 7 | 13.0 | 51.1 | 23.3 | 12.6 |  |
| 9 | 5.4 | 33.3 | 35.2 | 26.1 | 584.90*** |
| Relative family affluence |  |  |  |  |  |
| Lowest 20pct | 17.8 | 41.3 | 23.6 | 17.3 |  |
| Medium 60pct | 12.5 | 45.4 | 26.4 | 15.7 |  |
| Highest 20pct | 15.7 | 46.5 | 23.3 | 14.6 | 17.75** |
|  |  |  |  |  |  |
|  |  |  |  |  | ANOVA (F) |
| Psychosomatic complaints | 14.45 | 17.19 | 20.54 | 23.67 | 281.54*** |

*** p<0.001 **p<0.01 *p<0.05

**Table S3** Results from linear regression analyses of psychosomatic complaints regressed on school pressure, physical activity, and covariates. Models adjusting simultaneously for school pressure, grade, relative family affluence, and physical activity. Regression coefficients (b) and 95% confidence intervals (95% CI) with robust standard errors. Boys. n=1,827

|  | Crude^a^ | | Model 1^b^ | | Model 2^c^ | |
| --- | --- | --- | --- | --- | --- | --- |
|  | b | 95% CI | b | 95% CI | b | 95% CI |
| School pressure |  |  |  |  |  |  |
| Not at all (ref.) | 0.00 | - | 0.00 | - | 0.00 | - |
| A little | 2.18*** | 1.49, 2.87 | 2.23*** | 1.51, 2.96 | 2.20*** | 1.48, 2.93 |
| Some | 4.68*** | 3.82, 5.53 | 4.78*** | 3.84, 5.72 | 4.78*** | 3.82, 5.73 |
| A lot | 6.54*** | 5.47, 7.61 | 6.69*** | 5.52, 7.87 | 6.64*** | 5.45, 7.82 |
| Grade |  |  |  |  |  |  |
| 5 (ref.) | 0.00 | - | 0.00 | - | 0.00 | - |
| 7 | -0.02 | -0.82, 0.79 | -0.63 | -1.37, 0.11 | -0.63 | -1.36, 0.11 |
| 9 | 1.20** | 0.38, 2.02 | -0.48 | -1.32, 0.36 | -0.45 | -1.29, 0.38 |
| Relative family affluence |  |  |  |  |  |  |
| Lowest 20pct (ref.) | 0.00 | - | 0.00 | - | 0.00 | - |
| Medium 60pct | 0.42 | -0.40, 1.25 | 0.34 | -0.42, 1.11 | 0.43 | -0.32, 1.18 |
| Highest 20pct | -0.02 | -1.08, 1.04 | -0.10 | -1.08, 0.87 | 0.13 | -0.84, 1.10 |
| Physical activity |  |  |  |  |  |  |
| Once a month or less (ref.) | 0.00 | - |  |  | 0.00 | - |
| 1-3 days a week | -1.21* | -2.16, -2.27 |  |  | -0.91* | -1.79, -0.04 |
| 4 days a week or more | -1.57** | -2.55, -0.59 |  |  | -1.44** | -2.38, -0.51 |
|  |  |  |  |  |  |  |
| School pressure # Physical activity^d^ |  |  |  |  |  | p=0.384 |

***p<0.001 **p<0.01 *p<0.05

^a^ Crude analyses include one independent variable at the time.

^b^ Model 1 includes school pressure, grade, and relative family affluence.

^c^ Model 2 includes school pressure, grade, relative family affluence, and physical activity.

^d^ P-value from Wald test comparing the model fit between models without and with an interaction term between school pressure and physical activity.

**Table S4** Results from linear regression analyses of psychosomatic complaints regressed on school pressure, physical activity, and covariates. Models adjusting simultaneously for school pressure, grade, relative family affluence, and physical activity. Regression coefficients (b) and 95% confidence intervals (95% CI) with robust standard errors. Girls. n=1,918

|  | Crude^a^ | | Model 1^b^ | | Model 2^c^ | |
| --- | --- | --- | --- | --- | --- | --- |
|  | b | 95% CI | b | 95% CI | b | 95% CI |
| School pressure |  |  |  |  |  |  |
| Not at all (ref.) | 0.00 | - | 0.00 | - | 0.00 | - |
| A little | 3.21*** | 2.02, 4.40 | 3.01*** | 1.81, 4.21 | 3.06*** | 1.87, 4.25 |
| Some | 6.64*** | 5.45, 7.83 | 6.18*** | 4.93, 7.43 | 6.19*** | 4.95, 7.43 |
| A lot | 10.09*** | 8.83, 11.36 | 9.51*** | 8.17, 10.86 | 9.52*** | 8.18, 10.85 |
| Grade |  |  |  |  |  |  |
| 5 (ref.) | 0.00 | - | 0.00 | - | 0.00 | - |
| 7 | 2.21*** | 1.30, 3.12 | 0.44 | -0.41, 1.28 | 0.40 | -0.44, 1.25 |
| 9 | 4.23*** | 3.38, 5.09 | 1.01* | 0.18, 1.84 | 0.96* | 0.12, 1.81 |
| Relative family affluence |  |  |  |  |  |  |
| Lowest 20pct (ref.) | 0.00 | - | 0.00 | - | 0.00 | - |
| Medium 60pct | -0.15 | -1.09, 0.79 | -0.36 | -1.14, 0.42 | -0.26 | -1.06, 0.54 |
| Highest 20pct | -1.08 | -2.22, 0.05 | -0.79 | -1.72, 0.14 | -0.65 | -1.61, 0.31 |
| Physical activity |  |  |  |  |  |  |
| Once a month or less (ref.) | 0.00 | - |  |  | 0.00 | - |
| 1-3 days a week | -1.41** | -2.23, -0.58 |  |  | -0.73 | -1.52, 0.05 |
| 4 days a week or more | -1.41** | -2.36, -0.46 |  |  | -0.65 | -1.51, 0.22 |
|  |  |  |  |  |  |  |
| School pressure # Physical activity^d^ |  |  |  |  |  | p=0.141 |

***p<0.001 **p<0.01 *p<0.05

^a^ Crude analyses include one independent variable at the time.

^b^ Model 1 includes school pressure, grade, and relative family affluence.

^c^ Model 2 includes school pressure, grade, relative family affluence, and physical activity.

^d^ P-value from Wald test comparing the model fit between models without and with an interaction term between school pressure and physical activity.
